# Hyperimmune Plasma and Immunoglobulins against COVID-19: A Narrative Review

**DOI:** 10.3390/life14020214

**Published:** 2024-02-01

**Authors:** Massimo Franchini, Daniele Focosi

**Affiliations:** 1 Department of Transfusion Medicine and Hematology, Carlo Poma Hospital, 46100 Mantua, Italy; 2 North-Western Tuscany Blood Bank, Pisa University Hospital, 56124 Pisa, Italy; daniele.focosi@gmail.com

**Keywords:** COVID-19, SARS-CoV-2, convalescent plasma, hyperimmune immunoglobulins

## Abstract

Since late 2019, the new SARS-CoV-2 virus belonging to the *Coronaviridae* family has been responsible for COVID-19 pandemic, a severe acute respiratory syndrome. Several antiviral therapies, mostly derived from previous epidemics, were initially repurposed to fight this not rarely life-threatening respiratory illness. Among them, however, the only specific antibody-based therapy available against SARS-CoV-2 infection during the first year of the pandemic was represented by COVID-19 convalescent plasma (CCP). CCP, collected from recovered individuals, contains high levels of polyclonal antibodies of different subclasses able to neutralize SARS-CoV-2 infection. Tens of randomized controlled trials have been conducted during the last three years of the pandemic to evaluate the safety and the clinical efficacy of CCP in both hospitalized and ambulatory COVID-19 patients, whose main results will be summarized in this narrative review. In addition, we will present the current knowledge on the development of anti-SARS-CoV-2 hyperimmune polyclonal immunoglobulins.

## 1. Introduction

In late 2019, the world was faced with the discovery of a disease (called COVID-19) caused by a new virus, SARS-CoV-2 of the *Coronaviridae* family, responsible for a severe acute respiratory syndrome (SARS). According to the World Health Organization (WHO) coronavirus panel on COVID-19, confirmed cases worldwide as of November 2023 were more than 770,000,000 with nearly 7,000,000 confirmed deaths [1]. The peculiar mechanism of infection by SARS-CoV-2 (the virus enters the cell mostly through the cell membrane ACE2 receptor, which is distributed ubiquitously in human cells) accounts for the systemic involvement that, not rarely, causes severe multiorgan damage and an uncontrolled immune response with the release of pro-inflammatory cytokines (the so-called “cytokine storm”), which is involved in worsening the clinical conditions of the patients [2].

At the beginning of the COVID-19 pandemic, the issue of treatment of the frequently severe acute respiratory forms of SARS-CoV-2 infection emerged, representing a challenge for physicians operating in subintensive and intensive care units. In parallel with the repurposing of antiviral drugs already employed in previous epidemics (e.g., Ebola and HIV), the deployment of the collection of convalescent plasma against COVID-19 (CCP) started, based on the previous positive experience in other respiratory infections [2,3,4,5,6]. CCP, which contains high concentrations of polyclonal antibodies able to neutralize SARS-CoV-2 replication (neutralizing antibodies, nAbs), was the only specific antibody-based therapy available against SARS-CoV-2 infection during the first year of the pandemic and the most studied among the antiviral agents (over 30 randomized controlled trials, RCTs, conducted during the three years of the pandemic) [7]. Following the collection and the clinical use of CCP, the possibility of fractionating CCP to produce hyperimmune intravenous polyclonal immunoglobulins (hIVIg), a more concentrated plasma-derived product, against COVID-19 was investigated and deployed in several countries. The mutations of SARS-CoV-2 during the three years of the pandemic have led to the continuous creation of new viral variants that have made challenging the management of COVID-19. The aim of this narrative review is to critically summarize the role of CCP and hIVIg against COVID-19.

## 2. Search Methods

A literature search of PubMed (through Medline), EMBASE, Cochrane central, medRxiv and bioRxiv databases was carried out between 1 December 2019 and 14 November 2023, using English language as restriction. A literature search through MEDLINE and PubMed electronic databases was performed for publications during the period using the following Medical Subject Heading (MeSH) and keywords: “COVID-19”, “SARS-CoV-2”, “COVID-19 convalescent plasma”, “hyperimmune plasma”, “passive immunotherapy”, “therapy”, “hospital”, “outpatients”, “ambulatory”, “safety”, “randomized controlled trials” and “hyperimmune immunoglobulins”. We also screened the reference lists of the most relevant review articles for additional studies not captured in our initial literature search.

## 3. Convalescent Plasma

### 3.1. Characteristics of CCP

As previously mentioned, CCP was the only antibody-based anti-SARS-CoV-2 therapy available during the first year of the pandemic, up to the marketing of anti-Spike monoclonal antibodies (mAbs) (March 2021) [8,9]. Initially, CCP was generally collected by single productive plasmapheresis from patients recovered from SARS-CoV-2 infection, providing the negativity of serological and molecular markers of the main infectious diseases (i.e., HIV and hepatitis B and C infections plus negative molecular testing results of parvovirus B_19_, hepatitis A and E viruses, which were mandatory in many countries) and the pathogen reduction (i.e., photoinactivation including methylene blue + visible light, riboflavin + ultraviolet B and amotosalen + ultraviolet A) as a further security measure [10]. Donors’ age and hospitalization for severe COVID-19 were positively associated with greater antibody responses and higher levels in donated CCP units of nAbs [11]. Following the widespread diffusion of COVID-19 pandemic into the population, it was soon possible to collect CCP from regular blood donors, thus further increasing the CCP safety against known and unknown blood-borne pathogens avoiding the need for pathogen reduction technologies, which have been reported to potentially compromise Fc-mediated functions of nAb in CCP units [12]. In addition, the demonstration of the excellent correlation between the measurement of anti-SARS-CoV-2 antibodies using high-throughput automated serology and the measurement of nAbs with viral neutralization tests (time- and money-consuming, mandatory during the initial pandemic phase) provided a cheaper and faster solution to the biological validation issue of CCP. All these simplified measures render the CCP production from transfusion centers easier to perform. Notably, the mass vaccination campaign since early 2021 combined with the unrestricted wave of SARS-CoV-2 infection has led to an unprecedently high prevalence of high titers of nAbs in regular blood donors regardless of symptom severity. The resulting hybrid immunity has permitted the collection of the CCP from blood donors vaccinated and recently recovered from COVID-19. The so-called VAX-CCP currently represents a formidable and potent source of high-titer nAbs (having nAb titers over 10 times higher than those of regular CCP) with a preserved efficacy against circulating and, probably, future SARS-CoV-2 variants [13].

**Figure 1:**
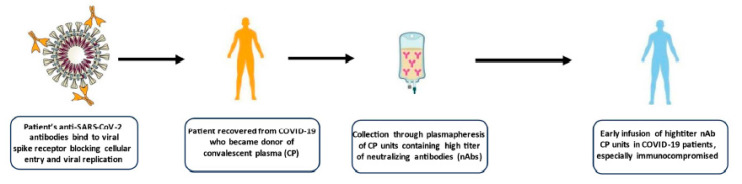
Mechanism of action, collection, and clinical indications of CCP.

Regarding the mechanisms of action of CCP, the effectiveness of anti-spike nAbs in blocking viral entry into host cells and the resulting viral replication has been well-characterized. Besides such activity, other anti-inflammatory properties of CCP have been recognized, helping in switching off the dangerous inflammatory process triggered by SARS-CoV-2. In addition, the inhibitory effects of anti-A natural isoagglutinin (contained in CCP from donors belonging to blood types O or B) that block viral attachment to cells (SARS-CoV-2 upholsters its envelope with A blood group antigens to escape host immunity) has been hypothesized by some investigators [14,15]. Figure 1 summarizes the collection, mechanism of action, and clinical indications for CCP against COVID-19.

### 3.2. Safety of CCP

As previously stated, much of the additional qualification tests initially mandated for CCP was proposed based on additional infectious safety concerns, which have proven unfounded so far. Additional concerns were initially raised, such as antibody-dependent enhancement (ADE) of viral infection, and transfusion reactions. All those concerns have remained theoretical so far, and several systematic reviews and metanalyses have found no increased risk compared with transfusion of fresh frozen plasma [16]. An additional concern regards the occurrence of venous and arterial thrombotic adverse events related to CCP transfusion, an issue not so trivial considering the highly prothrombotic context of COVID-19 and the presence of procoagulant factors in fresh frozen plasma. A recent systematic review and meta-analysis of 39 RCTs enrolling nearly 24,000 participants did not find an increased incidence of thromboembolic complications in CCP-treated patients versus controls [17], reassuring definitively regarding the safety profile of CCP against the thrombotic risk.

### 3.3. Clinical Indications of CCP

#### 3.3.1. Hospitalized Patients

Following the early positive experience in hospitalized COVID-19 patients from China and Italy [18], CCP was the object of intense research from investigators worldwide and, among anti-COVID-19 therapies, it has been the most extensively studied, being its safety and efficacy assessed by more than 30 randomized controlled trials (RCTs) and more than 100 non-RCTs so far, whose results have been fully published [19,20]. A strong inverse correlation between CCP use and mortality per admission in USA was observed during the winter 2020–2021 [21]. Overall, the evidence from the literature consistently supports the beneficial effect of CCP containing high anti-SARS-CoV-2 nAb (titer > 1:160) and administered within 3 to 5 days of infection [22]. In single-agent, placebo-controlled RCTs, delayed CCP use has generally been associated with no benefit [20]. However, a re-evaluation of literature data discovered signals of CCP efficacy even in unfavorable RCTs when subgroups of patients receiving early and high-titer CCP treatment were analyzed (Table 1) [23]. For instance, the multicenter Italian Transfusion of Convalescent Plasma for the Early Treatment of Patients With COVID-19 (TSUNAMI) trial, which was conducted in 487 hospitalized COVID-19 patients enrolled from 27 clinical centers, compared the effect of CCP with high titer nAbs (≥1:160) associated with standard therapy versus standard therapy alone in patients with COVID-19 and pneumonia with mild to moderate ventilatory impairment (defined from a PaO_2_/FiO_2_ ratio between 350 and 200) [24]. Overall, TSUNAMI did not show a plasma benefit in terms of reducing the risk of respiratory worsening or death in the first 30 days. However, the analysis of the different subgroups found a trend in favor of plasma in patients with less severe respiratory impairment (with a PaO_2_/FiO_2_ ratio ≥300 at enrollment). The trial was published in November 2021 but its negative results on CCP efficacy were anticipated in an international press release by the promoter on April 2021. This RCT had a detrimental effect on CCP for at least two reasons: first, it caused the stop of CCP use (and therefore the collection) not only in Italy but also in most western countries. Secondly, it ignored important signals of CCP efficacy in a subgroup of patients with mild-moderate COVID-19, which should have pushed the conduction in Italy of a further trial in COVID-19 patients in the early stages of the disease. The CAPSID RCT in Germany, which randomized 105 patients hospitalized for severe COVID-19 to receive standard treatment and CCP or standard treatment alone, found that CCP transfusion was not associated with a significant improvement in primary composite outcomes (survival and improvement of severe COVID-19 on day 21) [25]. A subgroup analysis, however, performed on patients who were provided CCP containing a higher cumulative amount of nAbs found a better survival and significantly shorter intervals to clinical improvement and to hospital discharge in CCP group in comparison with control group. In the CONTAIN RCT, which randomized 941 participants (473 to placebo and 468 to CCP), CCP did not meet the prespecified primary endpoint (clinical improvement at day +14 according to the WHO ordinal scale) although, in a subgroup exploratory analysis, a possible benefit of high-titer CCP was observed early in the pandemic [26]. In another RCT from Brazil, 107 patients with severe COVID-19 were randomized to receive CCP plus standard treatment or standard treatment alone [27]. Although no statistically significant reduction in mortality, requirement for invasive ventilation, and duration of hospital stay was observed between cases and controls, a trend towards a survival advantage at days +30 and +60 was signaled in the CCP arm. In a double-blind trial from USA conducted in 74 hospitalized COVID-19 patients who were randomized to receive CCP or standard plasma, the administration of CCP increased levels of antibodies against SARS-CoV-2 but was not accompanied by an improved outcome (evaluated as difference in ventilator-free days or mortality at day +28) [28]. Nevertheless, all-cause mortality through 90 days was numerically lower in the CCP group than standard plasma group (27% vs. 33%; *p* = 0.63). In the RCT by Li and colleagues, which enrolled 103 patients (52 in CCP plus standard therapy and 51 in standard therapy alone) with severe or life-threatening COVID-19, there was no significant difference in the primary outcome (time to clinical improvement within 28 days) in patients receiving CCP or not. Notably, a lower 28-day mortality rate (15.7% versus 24.0%) existed in the CCP group [29]. Although in the ConCOVID trial (86 patients: 43 treated with CCP and 43 with standard of care) the administration of CCP had no effect on disease course and did not significantly improved survival, a trend towards a CCP-related mortality reduction was noted (mortality in CCP of 14% versus 26% in the control group) [30]. In a recent French RCT recruiting 120 patients randomized to CCP (60 patients) or standard therapy (60 patients), CCP was not associated with a clinical improvement, but a trend towards reduction in mortality at days +14 and +28 in the CCP group was observed [31].

Besides the above-mentioned signals of CCP efficacy in many RCTs, a recent RCT showed a statistically significant mortality reduction in a subgroup of critically ill COVID-19 patients under invasive mechanical ventilation treated early (within 5 days from intubation) with CCP in comparison with placebo [32]. The interesting finding of the clinical efficacy of an antibody-based therapy in mechanically ventilated severe COVID-19 patients has been already observed in previous trials [28,29,30,33,34,35,36] and it is probably related to a SARS-CoV-2-induced early acute respiratory distress syndrome (ARDS) rather than to an inflammatory-related late ARDS.

Several systematic reviews have performed pooled analyses of the studies assessing the effectiveness of CCP therapy in hospitalized COVID-19 patients. A recent systematic review evaluated 39 RCT enrolling 21,529 participants and showed that CCP use in hospitalized patients was associated with a 13% reduced risk of mortality [37]. The mortality benefit was more evident when CCP contained high levels of nAbs and was administered within 5 days since hospitalization.

#### 3.3.2. Outpatients

A few RCTs have assessed the potential benefit of early CCP administration in ambulatory COVID-19 patients. The first was a double-blind RCT conducted in Argentina in a population of 160 elderly outpatients at risk for disease progression, and CCP reduced disease progression (16% in those who received CCP versus 31% in those who received standard care) [38]. In another double-blind RCT conducted in USA (CSSC-004), 1181 outpatients were randomly assigned to receive either high-titer CCP or placebo control plasma, and CCP administration led to a reduction in hospitalization within 28 days (2.9% versus 6.3%; *p* = 0.004) [39]. No statistically significant difference in symptom duration and resolution at day 14 was observed between the CCP-treated and control groups [40]. In a subsequent subgroup analysis of the CSSC-004 trial, among the 882 individuals with confirmed SARS-CoV-2 participating to study the association between CCP treatment, cytokine levels and post-COVID-19 conditions were investigated [41]. While in a multivariate analysis the female sex and the presence of elevated levels of interleukin-6 (IL-6) were independently associated with development of post-COVID-19 conditions (PCC), patients who received early CCP treatment (≤5 days after symptom onset) compared with late CCP treatment had statistically significant lower odds of PCC. A RCT run in the Netherlands (CoV-Early) randomized 421 outpatients aged 50 years or older at risk for progression to receive CCP or fresh frozen plasma. No benefit was detected in the entire cohort, but the effect of CCP on hospital admission or death was, although not statistically significant (odds ratio [OR] 0.66; 95% CI 0.394–1.085), largest in patients with 5 days or less of symptoms [42]. Similar negative results (no effect of CCP in preventing progression from mild to severe illness) emerged also in another RCT from Spain recruiting 376 COVID-19 outpatients randomized to receive high-titer methylene blue-treated CCP or placebo [43].

A meta-analysis of outpatient CCP treatment found a 3.7% absolute risk reduction (RR) and a 30.1% relative RR for all cause hospital admission. High-titer CCP transfusion, provided within 5 days of symptom onset, increased this to a 7.6% absolute RR and a 51.4% relative RR [44]. Another systematic review and meta-analysis, analyzing the determinants of passive antibody efficacy in SARS-CoV-2 infection, found a significant association between the dose administered and the efficacy in preventing hospitalization (RR 0.77; *p* = 0.0001) [45]. A recent metanalysis comparing the efficacy of CCP with other outpatient regimens has shown that CCP is only slightly inferior to authorized anti-Spike mAbs and small-molecule antivirals [46]. However, considering that anti-Spike mAbs have all been deauthorized in 2022–2023 because of the loss of baseline activity against recent Omicron sub-lineages, CCP remains the only passive immunotherapy available against COVID-19 [43]. In addition, having very few adverse reactions [17], CCP represents a valid alternative for this large group of frail patients who have contraindications or cannot tolerate the toxicities of small-molecule antivirals [47]. It must be outlined, however, that in many European countries CCP use has never been authorized (not even at an emergency level like in the US), and therefore it has always remained an experimental product only for in-hospital use within ethical committee-approved protocols. Such decision has irremediably damaged CCP efficacy, favoring studies on late hospital patients instead of those on early, and more appropriate, ambulatory patients. Thus, proper RCTs on CCP use in outpatients were conducted only in a late pandemic phase, following the demonstration of the efficacy of early use of anti-Spike mAbs against SARS-CoV-2.

#### 3.3.3. Immunocompromised Patients

The published literature data have consistently documented that, beside the high-titer nAb content and early administration, CCP exerts its maximum effect in seronegative patients, like immunocompromised patients (i.e., elderly subjects, patients with congenital or acquired immunodeficiencies, patients with solid or hematological cancer and recipient of organ transplants), who are not able to produce enough antibodies themselves in response to vaccination against SARS-CoV-2 or during COVID-19. In these fragile patients, even the more recent milder omicron variants of SARS-CoV-2 can replicate undisturbed, triggering the inflammation cascade and thus producing the sadly well-known life-threatening consequences [48]. The issue of the persistence of SARS-CoV-2 infection remains a major healthcare concern for immunocompromised subjects and is particularly relevant for onco-hematological patients such as those who are B cell depleted as a consequence of anti-CD20 mAb therapy, and are consequently not able to clear autonomously the virus. There is evidence that such patients may benefit from a longer duration of treatment with repeated CCP infusions, which are required to reach and maintain SARS-CoV-2 virus eradication. Evidence for the benefit of CCP in immunocompromised COVID-19 people is growing and systematic reviews have shown CCP efficacy in both primary and secondary immunodeficiencies. A 2023 systematic review and meta-analysis including 3 RCTs and 5 controlled studies in immunocompromised COVID-19 patients demonstrated that CCP decreased mortality compared with the control cohort (RR 0.63; 95% CI 0.50–0.79) [49].

Importantly, while no anti-Spike mAb or small molecule antiviral has been specifically investigated in RCTs in immunocompromised patients, this has been performed for CCP. A recent RCT employing Vax-CCP and enrolling 134 patients, showed that CCP significantly improved the survival in patients with hematologic or solid cancers (hazard ratio [HR] 0.28; *p* = 0.042) versus the standard of care arm. In addition, a subgroup analysis of the previously mentioned French RCT showed that CCP use was associated with mortality reduction (HR 0.39; 95% CI 0.14–1.10) [50]. Of note, a longitudinal cohort and propensity score analysis carried out by Hueso in patients with B-cell lymphoid neoplasm and COVID-19 showed that CCP significantly reduced mortality in patients who received anti-CD20 monoclonal antibody therapy [51]. As a consequence, a growing number of scientific societies and regulatory authorities around the world have acknowledged such efficacy by recommending CCP in their guidelines in immunocompromised patients with COVID-19 [52], with the exception of WHO and Cochrane reviews, which have not updated their recommendations on the basis of subgroup analyses [20].

## 4. Hyperimmune Immunoglobulins

As the pandemic evolved, several countries have begun to direct CCP donations towards plasma manufacturers to investigate CCP fractionation resulting in polyclonal IgG formulations [53]. The production on a large scale of anti-SARS-CoV-2 hyperimmune intravenous immunoglobulins (hIVIg), aimed at marketing a plasma-derived product with a high and standardized nAb titer against SARS-CoV-2 (hIVIG are approximately 10-fold more concentrated than CCP) in a smaller volume have encountered several obstacles. First, each lot of hIVIg is manufactured from CCP collected from a large number of donors (it is typically prepared from pools of 100–1000 L of CCP) and during the first pre-vaccine year of the pandemic it was very difficult to retrieve enough CCP donations because most of the CCP units collected were utilized for the emergency treatment of COVID-19, being the only antibody-based therapy available at that time. In addition, hIVIg manufacturing requires several months between initiation of CCP collection and distribution of lots (creation of dedicated CCP production chains according to Good Manufacturing Practice [GMP] rules, conduction of well-designed phase I–III trials and the acquisition of all necessary certifications for marketing by regulatory agencies). Another issue of equal importance is the economic sustainability related to the high costs of production of IVIg, whose price is noticeably higher than that of CCP. This has induced many manufacturers to consider investments on hIVIg economically non-advantageous as requiring several years (well-beyond the duration of the pandemic) to be amortized [53]. The results of RCTs on the clinical use of hIVIg against COVID-19 have been contradictory [53]. In a phase I/II RCT by Ali and colleagues in 50 patients with severe and critical COVID-19 and randomized to receive hIVIg or standard of care, the administration of anti-SARS-CoV-2 specific immunoglobulins was associated with a mortality reduction (25% in the intervention group versus 60% in the control group) [54]. The administration of hIVIg in 60 hospitalized COVID-19 patients, randomized to receive hIVIg or standard care, was found to be safe and well-tolerated, being characterized by respect to the control arm by a shorter time to viral clearance and an early reduction in inflammatory biomarkers [55]. Another RCT enrolled 18 severely immunocompromised patients hospitalized for COVID-19 to receive either hIVIg (10 patients) or standard IVIg (8 patients) and showed that hIVIg significantly reduced the incidence of severe COVID-19 (20% versus 88%, *p* = 0.015) [56]. By contrast, the ITAC RCT enrolled 593 participants of whom 301 received hIVIg an 292 placebo [57]: compared with placebo, the hIVIg group did not have significantly greater odds of a more favorable outcome at day 7 (adjusted OR: 1.06; 95% CI 0.77–1.45; *p* = 0.72). A recent Cochrane systematic review identified 5 RCTs with 947 participants (688 treated with different formulations of hIVIg) hospitalized with moderate or severe COVID-19 [58]. With the limitations derived by the great heterogeneity (difference in dosing and human or animal origin) of marketed hIVIg formulations, the author found no impact of this treatment on patients’ mortality or clinical improvement. Notably, in a recent head-to-head comparative RCT (hVIg versus CCP), treatment outcomes were better in patients treated with hIVIg on day 28 but not on day 14 [59]. Overall, although limited, the available literature data seem to indicate that hIVIg have no beneficial effect in immunocompetent hospitalized COVID-19 patients, but a potential role in improving outcome in those severely immunocompromised. In the current post-vaccine era, however, the hybrid (i.e., vaccine and infection) exposure of large part of the population has led to high-titer heterologous immunity against SARS-CoV-2 providing an efficient cross-protection against most variants, including Omicron sub-lineages. Within this new context, currently available standard IVIg, originating mostly from CCP donations, equate hIVIg in term of anti-SARS-CoV-2 nAb content, thus rendering the creation of a dedicated manufacturing chain poorly cost-effective and obsolete.

## 5. Conclusions

Among anti-COVID-19 therapies, CCP has been the most extensively studied during the three years of the pandemic [60,61,62,63,64,65,66,67,68,69,70,71,72,73,74,75,76,77,78,79,80,81,82,83,84,85,86]. Thus, after more than 30 RCTs, it can be definitively concluded that CCP is safe and effective in reducing progression of COVID-19 when it contains high titers (>1:160) of SARS-CoV-2 nAbs and is administered within 3 to 5 days of infection, especially among seronegative patients who are unable to mount a sufficient antibody response following SARS-CoV-2 infection or vaccination, such as immunocompromised patients. Furthermore, it should be outlined that CCP, being transfused very closely to the time of collection, is currently the only passive antibody-based therapy active against the more recent omicron variants. Thus, considering this fact and that not rarely antiviral drugs (i.e., remdesivir and Paxlovid) are contraindicated in hospitalized patients due to comorbidities or concomitant therapies, CCP collection and use should be currently endorsed by health authorities and scientific societies. In addition, the current availability of VAX-CCP permits to have a very potent source of nAbs against Omicron sublineages which render this biological product particularly useful for the clinical application in immunosuppressed patients.

Although this review provides the most recent and updated information on CCP clinical use, some grey areas persist on CCP representing the basis for future research. For instance, the role of naturally occurring anti-A antibodies in CCP from O or B blood type donors in blocking SARS-CoV-2 replication and disease progression is currently unknown and deserving further investigation by experimental and clinical studies. In addition, although the relationship between the amount of high nAb titers and the clinical response has been ascertained by several trials, the exact therapeutic dose of anti-Spike antibodies in CCP therapy remains undefined: it is believed to result from the combination of nAb titers, cumulative CCP volume, and recipient’s body weight, but a systematic investigation has never been performed. Beside the already mentioned efficacy of high-titer CCP administered early (i.e., in outpatients or during the first days of hospitalization), the recent finding of the CCP efficacy in intubated COVID-19 patients is intriguing and opens new treatment scenarios and will be certainly the object of research in the next future, considering the paucity of effective treatments in such critical setting.

Further RCTs are also needed to assess CCP in combination regimens (i.e., with remdesivir or other antivirals) against newer viral variants or its effect in reducing long-term sequelae of COVID-19, considering the preliminary positive findings from a recent RCT [38]. It remains to be established whether, at the steady state of viral evolution, concentrated polyclonal IgG formulations would be equally effective as CCP.

## Figures and Tables

**Table 1:** Signals of CCP efficacy in RCTs conducted in hospitalized COVID-19 patients.

Study, Year [Reference]	Cases/Controls	Results	Signs of Efficacy
TSUNAMI, 2021 [24]	487 patients (241 CCP + ST/246 ST)	The primary end point occurred in 59 of 231 patients (25.5%) treated with CCP + ST and in 67 of 239 patients (28.0%) who received ST (OR, 0.88; 95% CI, 0.59–1.33; *p* = 0.54).	In patients with COVID-19 at an early stage at baseline, the primary end point occurred less frequently in the group treated with CP plus ST (8 of 69 [11.6%]) vs those who received ST (16 of 73 [21.9%]) (OR 0.47; 95% CI, 0.19–1.18; *p* = 0.059).
CAPSID, 2021 [25]	105 patients (53 CCP + ST/52 ST)	The primary end point occurred in 43.4% of patients in the CCP + ST group and in 32.7% of patients in the ST group (*p* = 0.32).	In the subgroup that received a higher cumulative amount of nAbs, significantly shorter intervals to clinical improvement (20 vs. 66 days, *p* < 0.05) and to hospital discharge (21 vs. 51 days, *p* = 0.03) and better survival (day-60 probability of survival 91.6% vs. 68.1%, *p =* 0.02) were observed in comparison with the control group.
CONTAIN, 2022 [26]	941 patients (468 CCP/473 placebo)	The cumulative adjusted OR (caOR) for the primary outcome was 0.94 (95% CI, 0.75–1.18).	A possible benefit of CCP was observed in the subgroup of patients treated during the first pandemic wave (April–June 2020) when steroids and remdesivir where not in use (caOR 0.72; 95% CI 0.46–1.13).
RBR-7f4mt9f, 2022 [27]	107 patients (36 CCP + ST/71 ST)	No statistically significant reduction in mortality, requirement for invasive ventilation, and duration of hospital stay was observed between cases and controls.	At day 30, death rates were 22% for CCP group and 25% for control group; at day 60, rates were 31% for CCP and 35% for control.
Bennet Guerrero, 2021 [28]	74 patients (59 CCP/15 SP)	No difference in ventilator-free days or mortality (27% vs. 33%) was observed at day 28 in CCP group versus SP group.	All-cause mortality through 90 days was numerically lower in the CCP group than standard plasma group (27% vs. 33%; *p* = 0.63).
Li, 2020 [29]	103 patients (52 CCP + ST/51 ST)	No significant difference was observed in time to clinical improvement within 28 days between CCP and control groups.	A 8.3% (15.7% versus 24.0%) absolute difference in mortality rate at day + 28 was observed in favor of CCP treated patients.
ConCOVID, 2023 [30]	86 patients (43 CCP/43 ST)	CCP had no effect on the disease course and did not improve survival.	Mortality in CCP group was 14% (6 out of 43) vs 26% in control group (11 out of 43) (OR, 0.47; 95% CI 0.15–1.38).
Lacombe, 2023 [31]	120 patients (60 CCP/60 ST)	No difference in early outcomes between CCP and standard care group was observed.	The survival rate at day +14 and day +28 was higher in the CCP group than in standard care group (mortality rate: 5% versus 13% at day +14 and 12% versus 20% at day +28).
